# A Data-Driven Approach to Defining Risk-Adjusted Coding Specificity Metrics for a Large U.S. Dementia Patient Cohort

**DOI:** 10.3390/healthcare12100983

**Published:** 2024-05-10

**Authors:** Kaylla Richardson, Sankari Penumaka, Jaleesa Smoot, Mansi Reddy Panaganti, Indu Radha Chinta, Devi Priya Guduri, Sucharitha Reddy Tiyyagura, John Martin, Michael Korvink, Laura H. Gunn

**Affiliations:** 1 Department of Public Health Sciences, University of North Carolina at Charlotte (UNC Charlotte), Charlotte, NC 28223, USA; kricha92@charlotte.edu (K.R.); jsmoot3@charlotte.edu (J.S.); 2 School of Data Science, University of North Carolina at Charlotte (UNC Charlotte), Charlotte, NC 28223, USA; ppenumak@charlotte.edu (S.P.); mpanagan@charlotte.edu (M.R.P.); ichinta@charlotte.edu (I.R.C.); dguduri@charlotte.edu (D.P.G.); stiyyagu@charlotte.edu (S.R.T.); 3 ITS Data Science, Premier, Inc., Charlotte, NC 28277, USA; john_martin@premierinc.com (J.M.); michael_korvink@premierinc.com (M.K.); 4 School of Public Health, Faculty of Medicine, Imperial College London, London W6 8RP, UK

**Keywords:** coding specificity, ICD-10, dementia

## Abstract

Medical coding impacts patient care quality, payor reimbursement, and system reliability through the precision of patient information documentation. Inadequate coding specificity can have significant consequences at administrative and patient levels. Models to identify and/or enhance coding specificity practices are needed. Clinical records are not always available, complete, or homogeneous, and clinically driven metrics to assess medical practices are not logistically feasible at the population level, particularly in non-centralized healthcare delivery systems and/or for those who only have access to claims data. Data-driven approaches that incorporate all available information are needed to explore coding specificity practices. Using N = 487,775 hospitalization records of individuals diagnosed with dementia and discharged in 2022 from a large all-payor administrative claims dataset, we fitted logistic regression models using patient and facility characteristics to explain the coding specificity of principal and secondary diagnoses of dementia. A two-step approach was produced to allow for the flexible clustering of patient-level outcomes. Model outcomes were then used within a Poisson binomial model to identify facilities that over- or under-specify dementia diagnoses against healthcare industry standards across hospitalizations. The results indicate that multiple factors are significantly associated with dementia coding specificity, especially for principal diagnoses of dementia (AUC = 0.727). The practical use of this novel risk-adjusted metric is demonstrated for a sample of facilities and geospatially via a U.S. map. This study’s findings provide healthcare facilities with a benchmark for assessing coding specificity practices and developing quality enhancements to align with healthcare industry standards, ultimately contributing to better patient care and healthcare system reliability.

## 1. Introduction

The precise recording, evaluation, and documentation of patient information through medical coding play a large role in the quality of care delivered, reimbursement from payors, and the reliability of healthcare systems [1]. The process of clinical coding has multiple facets, such as accuracy, completeness, and appropriate levels for the specificity of diagnostic coding. Coding specificity, an important aspect of the coding process, refers to the level of granularity at which a clinical diagnosis is recorded [2].

A widely used medical coding system is the International Classification of Diseases Clinical Modification (or ICD-CM) [3]. ICD-10-CM (herein ICD-10) refers to the 10th revision of the taxonomy and serves to categorize diseases and health conditions at varying degrees of specificity, from coarse or unspecified more general diagnoses to more granular ones representing a deeper level of knowledge of the clinical condition. Medical coding helps standardize documentation across healthcare systems, serves as a tool for medical billing, standardizes risk factors in risk-adjusted quality measures, and helps guide healthcare policy by accurately identifying disease prevalence and supporting national and international decision making [3]. The coding system also helps organize and find medical information easily, impacting how we understand and use medical data, including assessing in real time the spread or prevalence of diseases and the optimization of resource allocations [3,4,5,6].

When considering coding practice enhancements, it is important to assess how healthcare professionals in hospitals document the presence of a specific condition or disease. Transcription—potentially including voice transcription for electronic health records (EHRs)—errors, missing information in patient charts, and illegible handwriting all contribute to inadequate specificity in coding [7]. It constitutes a shared responsibility among all parties involved to appropriately code to the highest level of specificity [8]. The move from ICD-9 to ICD-10 on 1 October 2015 led to a fivefold increase in the number of codes, exacerbating the complexity of coders’ work and the potential for coding errors following this transition [3]. Clinical specialties have been affected differently, with this ICD-10 transition representing an uneven burden across facilities and patients depending on the diagnosis family and the facility’s level of specialization [9,10].

A lower coding specificity is a potential burden for Medicare and other payors, which are billed for potentially under-specified diagnoses when a higher specificity (i.e., enhanced diagnosis quality) could be available [3,8]. Medicare is the health insurance coverage provided by the United States (U.S.) government for individuals 65 years and older. Medicare within the acute care inpatient setting refers to payments reimbursed through the Inpatient Prospective Payment System (IPPS). Through the IPPS, hospitalizations are grouped into medical severity diagnosis-related groups (MS-DRGs) based largely on the presence of principal and secondary ICD-10-CM diagnosis codes, and in some cases, ICD-10-PCS procedure codes. The MS-DRG grouping is associated with a weight that further adjusts the base hospital payment (determined by a set of hospital-level characteristics) to determine the final discharge-level reimbursement. There is a tradeoff between productivity and coding quality, as enhancing the coding specificity can be time intensive in some instances, requiring the coder to explore whether more specific coding is appropriate given the information provided in the patient medical record. At the facility level, the associated costs to the healthcare payor as well as the impact on patients’ clinical history, treatment, and resulting health outcomes must also be considered [8]. Coding specificity is especially relevant when a diagnosis is unrelated to the principal cause of hospitalization, or when diagnoses are not made by specialists, as added specification for these codes may not affect the hospital’s rate of reimbursement for the hospitalization. Thus, coding specificity is not only important at the administrative level but also has the potential to impact both healthcare facilities and patients.

At the facility level, coding that accurately captures clinical diagnoses ensures that healthcare facilities maintain effective billing operations. Coding specificity has the potential to impact a facility’s financial capital and allocation of resources, since facilities, after a short grace period that ended in October 2016, can be denied Medicare-based claims based on insufficient diagnostic specificity [8,11]. ICD-10 codes are the foundation of hospital billing processes, so misdiagnoses or misclassifications of codes can impact hospital reimbursement and insurance eligibility, including Medicare reimbursements [12]. Inaccurate coding further has the potential to affect facilities’ reputations and pay-for-performance incentive payments, as such hospital-ranking programs evaluate quality performance using risk-adjusted outcomes that rely on ICD-10 coding [13,14,15,16,17,18].

From the patient perspective, accurate documentation ensures that the patient is receiving an adequate treatment plan tailored to the specifics of their diagnosis and needs, both during their inpatient stay and after discharge [8]. In addition to individuals who may go undiagnosed, those with an under-specified diagnosis may also suffer worsened clinical outcomes. Among other factors, practitioners’ unconscious biases as well as inappropriate facility practices could result in deviations from universal documentation and coding standards, thus potentially exacerbating health disparities [19], which may manifest through specificity gaps across subpopulations. A lack of specificity can lead to patients’ clinical histories being affected, thus resulting in potential variations in care and resulting outcomes across social strata [8]. From the standpoint of coding specificity, the literature lacks a wider understanding of how decreased (or increased) levels of specificity may be associated with sociodemographic factors, thus potentially exacerbating the aforementioned healthcare disparities [19].

Not all unspecified diagnoses are inappropriate. In fact, unspecified diagnosis codes are recommended by the United States (U.S.) Centers for Medicare and Medicaid Services (CMS): “*When sufficient clinical information is not known or available about a particular health condition to assign a more specific code, it is acceptable to report the appropriate unspecified code*” [20]. Hence, there is a balance between the necessary level of unspecificity and the unnecessary level of unspecificity that needs to be considered, since a pure minimization of unspecified codes could also lead to incorrectly specified diagnoses. Conversely, achieving a higher level of specificity may require additional clinical tests or interventions, which may be subject to additional considerations regarding cost-effectiveness [21,22,23], especially when the primary cause of the inpatient stay is not related to nor affected by the unspecified diagnosis. Also, higher levels of specificity may not be warranted by clinical diagnoses. Incorrect levels of both specificity and unspecificity can lead to inappropriate treatment. Thus, when a diagnosis is not confirmed, it is appropriate to provide an initial, temporary unspecified diagnosis [20] until further tests can be performed, if clinically recommended.

While our approach is generalizable and can be applied across clinical strata, our motivating example consists of a large patient cohort across the U.S. of nearly 500,000 unique inpatient individuals who were diagnosed with dementia and discharged in 2022. In 2020, dementia affected the lives of over 55 million people across the world, which is close to 1% of the global population [24]. Projections suggest that this number will experience nearly twofold growth every 20 years, surging to 78 million by 2030 and about 139 million by 2050 [24]. This number is further increased by those providing caregiving and other family members indirectly suffering from this debilitating disease. In the absence of enhanced treatments or preventive measures, adverse outcomes associated with dementia will persistently rise [25]. Many patients are likely to receive unspecified dementia diagnoses when seeing a primary care provider compared to when seeing a specialized provider (like a neurologist or geriatrician) [26,27]. Thus, dementia represents an important disease within an aging population that is likely to be of increased relevance as treatment interventions are developed, and enhanced coding specificity is needed in this area to identify resources properly [25]. In a 2017 study, researchers reviewed the medical records of dementia ICD-10 code cases, and they discovered that many of the cases lacked specific descriptions that would aid in confirming the diagnosis of specific types of dementia [28]. This study revealed that 63% of cases did not provide a specific diagnosis of dementia in the medical records, but instead considered other conditions as the likely explanation of the patient’s hospitalization [28]. More generally, mental health conditions have been identified among conditions suffering from higher rates of unspecified diagnoses [8].

Models have been developed for assessing risk-adjusted coding intensity for both diagnoses and procedures, as well as identifying facilities that over- or under-code [29,30]. This area tangentially relates to coding specificity. However, the literature still lacks risk-adjusted approaches that account for factors potentially associated with coding specificity, adjusting for patient and facility characteristics, with only some initial work developed in the area of depression [31], but none, to our knowledge, in the area of dementia or other neurocognitive diseases. The aim of this study is to provide a novel risk-adjusted metric, demonstrated through a population-based dementia patient cohort in the U.S., to estimate dementia ICD-10 coding specificity by facility upon adjusting for a set of commonly available facility- and patient-level characteristics.

While enhancements in coding specificity practices are possible through other means, such as through the clinical identification of potential coding specificity inaccuracies or increased training, such approaches are not cost-effective if they need to be performed at the population level. There is a need for cost-effective approaches that serve to pre-screen and identify facilities which may need such enhancements the most. Clinical assessments may be possible if electronic health records are available, but this is not always the case. In this case, data-driven approaches may provide insights into how coding specificity can vary across patients and facilities and whether these variations occur in ways that may depart from anticipated randomness. Our proposed data-driven metric can serve facilities to self-assess variation in coding specificity compared with their healthcare peers and can provide a benchmark to identify facilities that could benefit from a further analysis of diagnostic coding specificity practices.

## 2. Materials and Methods

### 2.1. Data and Variables

De-identified data sourced and provided by Premier, Inc.’s private database serve as the foundation of this analysis [32]. The dataset is composed of N = 487,775 observations containing information on the first inpatient hospitalization for each patient with a principal or secondary diagnosis related to dementia who was discharged in the year 2022 using the F ICD-10 diagnosis codes provided in Supplementary Tables S1 and S2. The ICD-10 codes corresponding to these diagnoses were identified by an expert team of medical coders at Premier, Inc. Patients who were admitted prior to 2022 were also included if they were discharged in 2022.

The data were further categorized into three types of variables: (1) outcome variables; (2) patient characteristics; and (3) facility characteristics. Outcome variables include coding specificity of principal diagnosis of dementia codes and coding specificity of secondary diagnosis of dementia codes. Principal diagnosis specificity denotes whether the ICD-10 dementia-related principal diagnosis code was specified (versus unspecified), and for secondary diagnoses, a specified diagnosis is assumed when at least one secondary diagnosis related to dementia was specified. In addition to masked patient IDs, patient characteristics for this study include the following: age group; sex; race; length of stay; primary payor; point of origin; discharge status; number of procedure codes; ICD-10 coding period (2022 for coding prior to 1 October 2022 and 2023 for codes from 1 October 2022); five Centers for Disease Control and Prevention’s Agency for Toxic Substances and Disease Registry’s (ATSDR) social vulnerability indices [33]; a COVID-19 indicator; and Medicare Severity Diagnosis Related Group (MS-DRG) type for the inpatient stay. In addition to masked facility IDs, facility characteristics include the following: three facility status variables (teaching, academic, and urban); ownership; size (bed count, grouped); case mix index (CMI); and U.S. state.

### 2.2. Statistical Analysis

Descriptive statistics were calculated and tabulated. Variables for which certain subgroups had limited representation (e.g., charity and indigent payors) were grouped together. Patients under 45 years old were grouped together due to their low counts. Discharge status codes indicating that the patient expired were collapsed into a single category. A diverse set of categories representing patients’ points of origin with low counts were grouped into a single ‘other’ category.

Univariate and multivariate logistic regression analyses were utilized to identify associations between patient- and facility-level characteristics and each of the two outcomes (specificity of dementia principal and secondary diagnoses per patient hospitalization). Univariate and adjusted odds ratios (ORs), as well as corresponding 95% confidence intervals (Cls) and *p*-values, were computed and tabulated. Receiver operating characteristic (ROC) curves were calculated and depicted, and area under the curve (AUC) values were extracted to demonstrate the multivariate models’ fitted performances to explain principal and secondary dementia diagnoses.

Clustering of this metric is demonstrated at the facility level, though other clustering factors are possible. Importantly, as opposed to variables used for constructing the patient-specific metric, clustering variables do not need to be observable for the full sample. A facility-specific metric of diagnostic coding specificity was also calculated from the risk-adjusted probabilities of specificity. Let $Y_{i,j}$ be the binary variable denoting coding specificity of the principal or secondary diagnosis for hospitalization *i* at facility *j*. This variable follows a Bernoulli (Ber) distribution with estimated probability ${\hat{p}}_{i,j}$ as shown below:

(1) $$Y_{i,j}\;\sim \;Ber\left({\hat{p}}_{i,j}\right).$$

The set ${\hat{p}}_{i,j}$ was estimated from the multivariate logistic regression model which was adjusted for patient and facility characteristics. Assuming that each hospitalization’s coding specificity was independently, though not identically, distributed per facility, the total count of facility-specific coding specificity follows a Poisson binomial (PoiBin) distribution with probability vector ${\hat{p}}_{j}=\left({\hat{p}}_{1,j,}{\hat{p}}_{2,j},\ldots ,{\hat{p}}_{n_{j},j}\right)$ for $n_{j}$ hospitalizations in facility *j* as shown as follows:

(2) $$\sum_{i=1}^{n_{j}}Y_{i,j}\;\sim \;PoiBin\;\left({\hat{p}}_{j}\right)$$

Facility-specific 95% CIs were extracted through the Poisson binomial facility-specific cumulative distribution functions (CDFs). These were used to identify facilities which under- (*p* < 0.025) and over- (*p* > 0.975) specified in their coding versus facilities’ peers using the estimated CDF for the specificity count.

Error bars were constructed to demonstrate the facility-specific metric for a sample of 20 facilities for both dementia principal and secondary diagnoses. Among these facilities, the coding specificity of dementia diagnosis indicator variable was defined, and an observed count (dots) was plotted for each facility (X-axis). A 95% CI for each facility, built on the basis of the Poisson binomial model, was added to identify these facilities’ adjusted levels of coding specificity against peers. Over- and under-coding risk-adjusted specificity practices were then identified by the facility.

Finally, geospatial U.S. maps were created to display adjusted ORs of principal and secondary diagnosis coding specificity by state against the reference of New York, which is the state with the highest per capita healthcare expenditure in the U.S. [34].

## 3. Results

Table 1 provides a summary of the descriptive statistics for N = 487,775 hospitalization records and patients, since each patient is only observed once due to the cohort definition (the first hospitalization for each patient within the year). The dataset comprised observations from 866 facilities, with an average of 563.25 patients per facility. The distribution of age among this dementia patient cohort is naturally skewed, with 61% of individuals being 80 years and older. Females constituted 58% of the patients, and the majority of the patients identified as White (76%). The median length of stay, which was log-transformed due to its large right skewness, was 5 days, and the most common primary payor was Medicare traditional (53%). The point of origin was predominantly non-healthcare facilities (79%), and the discharge status varied, with 19% of the patients being discharged to home or self-care, while the majority were transferred to other healthcare facilities, often skilled nursing facilities (36%). The average number of procedures during inpatient stays was 2.7, with surgical MS-DRGs representing 15% of hospitalizations. Additionally, 13% were COVID-19-positive patients. Most of the facilities were non-teaching (78%) and non-academic (85%). Urban facilities were more prevalent (86%) than rural ones. Voluntary non-profit private was the most common ownership status (64%). The bed capacity varied, with 1–50 beds (3%) and >400 beds (39%) being the least and most common facility sizes, respectively. The mean case mix index was 1.7. The dataset represented multiple states, with New York (9%) and Florida (12%) being the top states in the number of hospitalizations.

Table 2 contains the adjusted ORs, 95% CIs, and *p*-values for the univariate and multivariate logistic regression analyses for modeling the coding specificity of dementia-related principal diagnoses. Younger patients were generally associated with higher odds of coding specificity than patients in the oldest age group (85+). Males experienced 45% higher odds of dementia-related principal diagnosis coding specificity than females (OR = 1.454; 95% CI: 1.301–1.625). Race was generally non-significant, except for Black patients, who experienced significantly higher odds of principal diagnosis coding specificity than White patients (OR = 1.237; 95% CI: 1.058–1.446). The log-length of stay was significant, with longer stays associated with higher odds of coding specificity (OR = 1.124; 95% CI: 1.060–1.191), but primary payor and point of origin were generally not significant. Patients with certain discharge statuses experienced significantly higher odds of coding specificity than those discharged to home or self-care, namely patients discharged to hospice homes, hospice medical facilities, or psychiatric hospitals (OR ≥ 1.354). The number of procedures was also significant, with each additional procedure performed associated with 23% increased odds of coding specificity (OR = 1.230; 95% CI: 1.179–1.283). The CMS fiscal year was highly significant, indicating 73.6% lower odds of specificity for 2023 (discharges occurring between 1 October and 31 December 2022) compared to 2022 (discharges between 1 January and 30 September 2022) (OR = 0.264; 95% CI: 0.224–0.311). Social vulnerability indices were not significant at the multivariate level, though some were significant univariately, indicating that some of the information content may be present in other patient characteristics. COVID-19 status and MS-DRG type were not statistically significant, except for at the univariate level, at which the latter showed surgical MS-DRGs associated with increased odds of specificity. At the facility level, patients in facilities whose teaching status was not available experienced 65.1% lower odds of specificity than those in non-teaching facilities (OR = 0.349; 95% CI: 0.142–0.856). Neither academic nor rural/urban status showed significant variability at the multivariate level. Most ownership categories were not significantly different from the voluntary non-profit private reference, except for other non-profit voluntary (OR = 0.605; 95% CI: 0.442–0.827) and local government (OR = 2.104; 95% CI: 1.401–3.159). Patients from facilities with bed counts lower than the reference category (>400) experienced lower odds of coding specificity, though only three categories were statistically significant. The case mix index was significant, with each unit increase accompanied by 57.9% increased odds of dementia-related principal diagnosis coding specificity (OR = 1.579; 95% CI: 1.188–2.100). Finally, most states demonstrated no statistically significant differences in principal diagnosis coding specificity compared to New York, with the exception of Hawaii, Louisiana, Minnesota, Oregon, Pennsylvania, and Virginia (which had a higher odds) as well as Illinois and Tennessee (which had lower odds of coding specificity).

Table 3 reports the univariate and multivariate logistic regression results (ORs, 95% CIs, and *p*-values) for the specificity of secondary dementia diagnoses’ outcome. For the multivariate results, all age groups experienced higher odds of specificity of dementia secondary diagnoses than the reference group of ages 85+ (OR ≥ 1.316; *p* < 0.001). Male patients had significantly higher odds of dementia secondary diagnosis specificity compared to females (OR = 1.224, 95% CI: 1.209–1.239; *p* < 0.001). Individuals identifying as Black were associated with lower odds of dementia secondary diagnosis specificity (OR = 0.955; 95% CI: 0.937–0.973) compared to White patients, while the opposite was found for those identifying as other races (OR = 1.069; 95% CI: 1.040–1.099). For some categories, primary payor, patient origin, and discharge status also showed significant associations with dementia secondary diagnosis coding specificity (see Table 3). Length of stay (in log terms) was also associated with higher odds of dementia secondary diagnosis specificity (OR = 1.017; 95% CI: 1.008–1.025). Those undergoing a larger number of procedures experienced higher odds of dementia secondary diagnosis specificity (OR = 1.039; 95% CI: 1.036–1.042). The CMS fiscal year was not substantially different, with those who were hospitalized in the new 2023 fiscal year experiencing 1.4% higher odds of specificity (OR = 1.014; 95% CI: 1.000–1.028). Patient socioeconomic (OR = 0.829; 95% CI: 0.717–0.958) and racial/ethnic minority (OR = 1.09; 95% CI: 1.03–1.154) statuses within the social vulnerability indices were significantly associated with decreased and increased, respectively, odds of dementia secondary diagnosis specificity. COVID-19-positive patients were associated with lower odds of dementia secondary diagnosis specificity (OR = 0.948; 95% CI: 0.930–0.965). Patients undergoing a surgical MS-DRG experienced 14% lower odds of dementia secondary diagnosis specificity compared to those undergoing a medical MS-DRG (OR = 0.859; 95% CI: 0.844–0.875). Academic facilities demonstrated higher odds of dementia secondary diagnosis specificity (OR = 1.052; 95% CI: 1.020–1.085), whereas those in rural settings experienced lower odds of dementia secondary diagnosis specificity (OR = 0.976; 95% CI: 0.955–0.997). Patients at facilities of different ownership types also experienced differing odds of dementia secondary diagnosis specificity (see Table 3). Lower bed counts were generally associated with lower odds of dementia secondary diagnosis specificity (OR ≤ 0.954) than those in the largest cluster of hospitals (>400 beds), with the exception of facilities with 51–100 beds and those with 351–400 beds. Substantial differences in the odds of dementia secondary diagnosis specificity were found by state when compared to the reference state of New York.

**Figure 1:**
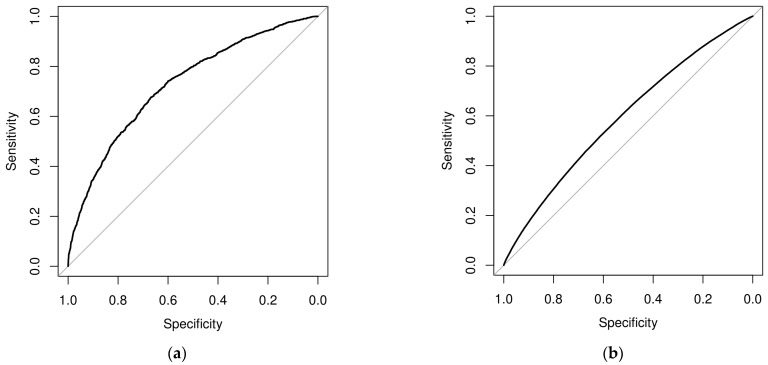
Receiver operating characteristic (ROC) curve of the multivariate logistic regression model for the specificity of a dementia-related principal diagnosis (**a**) and secondary diagnosis (**b**).

Figure 1 panel (a) shows the ROC curve for the multivariate model of the coding specificity of a principal diagnosis related to dementia. The estimated AUC was 0.7269, representing the good reliability of the multivariate model in assessing the coding specificity of dementia-related principal diagnoses. Panel (b) shows the ROC curve corresponding to the multivariate logistic regression analysis for assessing the coding specificity of secondary dementia diagnoses. The corresponding AUC was 0.5919, demonstrating a worse model performance when compared to that of the model assessing the coding specificity of primary dementia diagnoses.

**Figure 2:**
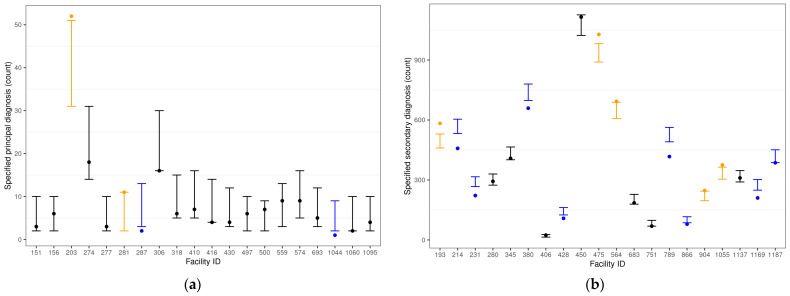
Observed counts of indicators of principal diagnosis coding specificity (**a**) and secondary diagnosis coding specificity (**b**) for dementia diagnoses by facility (dots) and 95% confidence intervals based on the Poisson binomial metric (error bars), with colors denoting over-specificity (orange), under-specificity (blue), and specificity in line with peers (black).

Figure 2 represents a subset of the facilities’ observed dementia-related principal diagnosis coding specificity (a) and secondary diagnosis coding specificity (b) relative to industry standards. The *p*-values (and the 95% CIs, which are represented as error bars) from the estimated Poisson binomial distribution are used so that under-specificity versus peers (*p* < 0.025) is represented in blue; specificity in line with peers (0.025 ≤ *p* ≤ 0.975) is represented in black; and over-specificity versus peers (*p* > 0.975) is represented in orange.

**Figure 3:**
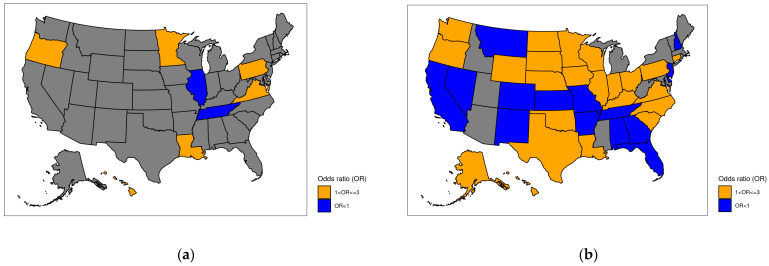
Geographical U.S. map of adjusted odds ratios (ORs) of coding specificity of dementia-related principal (**a**) and secondary (**b**) diagnoses by state, with a reference state of New York. Odds ratios that were not statistically significant are shown in gray.

Figure 3 represents the adjusted ORs for the coding specificity of a dementia-related principal diagnosis (a) and secondary diagnosis (b). All of the adjusted ORs are represented against New York as the reference state. Only a few states demonstrate statistically different adjusted odds of coding specificity of a dementia-related principal diagnosis versus New York, while a larger amount of variability is observed for states’ secondary diagnosis coding specificity. The gray states had non-significant adjusted ORs of coding specificity.

## 4. Discussion

The literature on diagnostic coding specificity remains scarce, with healthcare facilities and practitioners limited in their ability to self-evaluate against healthcare industry standards of practice. It is also unclear whether non-clinical characteristics can explain variability in specificity practices. To address this gap, a novel approach was demonstrated to evaluate facility-specific practices for the dementia-related coding specificity of principal and secondary diagnoses upon making risk adjustments for commonly available patient and facility characteristics. A logistic regression was applied to make risk adjustments to the probability of receiving a specified dementia diagnosis. The statistical output is used in a two-step approach, building on a Poisson binomial model, to evaluate the performance of healthcare facilities in providing specified dementia-related principal, or at least one secondary, diagnoses. This metric can be used to identify facilities that perform differently (under- or over-specifying) compared to their healthcare industry peers and can provide an objective standard against which the coding specificity practices of facilities can be evaluated. These findings offer valuable insights for healthcare stakeholders and quality-control personnel, facilitating the identification of facilities that may benefit from targeted interventions to enhance the levels of specificity of dementia-related diagnosis coding.

Our results indicate that the coding specificity of dementia diagnoses is associated with a range of patient and facility characteristics, particularly for primary diagnoses, as demonstrated through a higher AUC value. Younger patients were generally associated with a higher odds of coding specificity for dementia-related principal and secondary diagnoses. While dementia has been found to be more easily identifiable among older patients [35], our findings indicate that, conditional on a dementia diagnosis, the odds of coding specificity are higher among younger patients. However, it is unclear whether there is a clinical association between the prevalence of specified cases of dementia and age, particularly when comparing age groups with those at least 85 years old.

Prior studies have found that the prevalence of types of dementia is different by sex [36], which could also be due to environmental and behavioral differences according to sex. Males had approximately 22% (secondary) and 45% (principal) higher odds of dementia diagnosis specificity compared to females, though this could be confounded with age. Black patients demonstrated a significantly higher odds of principal diagnosis coding specificity than White patients. However, the reverse is observed for secondary diagnosis specificity. In both cases, there could be confounders due to collinearity with other factors, including social vulnerability indices. Patients have been shown to experience differences in the prevalence of dementia and its associated symptoms and severity by race [37], which could potentially have an association with the ability of doctors to provide a specified dementia diagnosis.

The significant association between longer hospital stays and higher odds of both principal and secondary coding specificity could be due to the additional inpatient time which allows for more comprehensive evaluations, diagnoses, and documentation. Patients discharged to specific destinations, such as hospice homes, hospice medical facilities, or psychiatric hospitals, exhibited significantly higher odds of principal and secondary diagnosis specificity. This could be related to the severity of their case or their prior history, which could, in turn, be associated with a potentially more accurate clinical diagnosis. Patients undergoing more procedures had higher odds of receiving a specified principal or secondary diagnosis. Though the cause of this association is unclear, this could be related to there being more resources allocated for identifying a patient’s disease when procedures are necessary during their inpatient stay. While a COVID-19 diagnosis was not associated with differing odds of principal diagnosis specificity, it was associated with lower odds of secondary diagnosis specificity. However, it is unclear whether the association between the severity of patients’ COVID-19 symptoms and age could be a confounder [38].

While the differences by CMS fiscal year in secondary diagnosis specificity were minor and are probably clinically irrelevant, the differences were more substantial among those with a dementia primary diagnosis. However, this could be due to seasonal confounders. The new fiscal year, denoted as 2023, was only measured in the October–December 2022 period, which may also be a period with seasonally over-burdened hospitals and less time for healthcare personnel to perform more in-depth diagnoses of patients.

From a payor perspective, none of the payor types were associated with differing odds of principal diagnosis specificity when compared to that of Medicare traditional. This is encouraging, as it indicates that principal diagnosis specificity may not be attributable to healthcare payor type. However, the substantial differences in the univariate results indicate that some complex associations may be embedded, though this is unclear, since the patient mix would not be homogeneous across payor types. For example, age could be acting as a proxy for Medicare status. Also, some differences were found when assessing odds of secondary diagnosis specificity. Some of these differences could be due to other patient characteristics. For example, those receiving Medicare traditional may be in widely different age groups than those for whom the payor comes from a direct employer contract or who receives workers’ compensation. Thus, health insurance coverage may be substantially different across patients, leading to the different propensities of patients to seek hospitalization [39].

Additionally, the ownership status of the facilities displayed some significant differences, with local government-owned facilities showing notably higher odds of principal and secondary diagnosis specificity. Again, the non-clinical patient characteristics by facility and facility ownership could differ widely. The case mix index of the hospital was significantly, positively associated with the specificity of principal diagnosis, indicating that the overall complexity of patients’ needs in a facility is related to higher degrees of specificity provided during a hospitalization. However, no significant association of specificity and the facility case mix index was found when the dementia diagnosis was secondary during the inpatient stay. Substantial differences were also found by state, particularly for secondary diagnoses. These differences could stem from the population mix or could be related to a substantially larger sample size for this analysis. Differences in health care provision by state across multiple metrics, such as care setting and type of disease/clinical area, have been documented [40]. However, we cannot link the coding specificity with the quality of care directly, since a low quality of care can occur when there are low levels of specificity state-wide but also when there are high levels of specificity and such excessive level of specificity is not clinically warranted.

These variations in coding practices demonstrate the potential influence of organizational characteristics or state-wide standards of practice on coding specificity. State-level variations may be attributed to regional variations in healthcare infrastructure, regulatory frameworks, insurance-related expectations/requirements, or coding practices. Also, there is state clustering of hospitals with a common health system, which may share a coding department and/or coding standards. However, they could also be influenced by the patient mix and other correlated factors in these states, given the socioeconomic, racial, and age differences across states, which may reflect the underlying reasons for non-idiosyncratic specificity disparities [41].

Providing high levels of coding specificity, when possible and appropriate, supports the accuracy and completeness of health records for patients, potentially enhancing their subsequent health outcomes. However, high coding standards require both time and educational/training resources for coders to conduct efficient and consistent coding practices that are current and accurate. Unspecified diagnoses may sometimes be a consequence of insufficient knowledge about all possible ICD-10 codes available related to a condition. Over-specified diagnoses may be a consequence of miscoding. Therefore, there is a tradeoff between the cost of specificity-related accuracy (oftentimes paid by the provider) and the cost of specificity-related inaccuracy (oftentimes a burden for the payor and the patient). Our approach demonstrates that facilities with dementia-related hospitalizations can be compared against a common/industry standard in a risk-adjusted form, so that facilities over- or under-specifying can be identified and their coding standards of practice can be adjusted, when needed.

While the proposed approach is demonstrated with an example of clustering at the facility level, for which full information is available for all patients, clustering by other factors is also possible. For example, clustering by zip code can allow for geospatial analyses of coding specificity. Also, clustering factors do not need to be available for all observations, allowing for more flexible analyses. For example, some hospitals may collect information about patients or systems that other hospitals do not collect. Clustering analyses are possible in such instances, and it is one of the core advantages of the two-step approach of performing patient-level analyses and subsequently clustering by any desired factor.

Our findings emphasize the association of multiple patient and facility characteristics with coding specificity. The relative significance of the evaluated variables in explaining the variability in coding specificity further underscores the importance of risk-adjusted performance metrics when comparing healthcare outcomes and facility performances.

### Strengths and Limitations

A large comprehensive dataset with nearly 488,000 patient observations related to dementia was used for this study, which represents, to our knowledge, the largest dementia-related study approaching the topic of diagnostic coding specificity. Developing and utilizing the proposed risk-adjusted metric allows for a fair assessment of coding specificity among healthcare facilities while producing an extrapolatable approach that allows for the incorporation of any available information about patient hospitalizations.

Though the dataset contains the most recently completed year (2022), it only encompasses a single year of discharges, yielding temporal limitations since coding policies and practices can be updated yearly. However, due to these potential dynamics, it is important to have a recent dataset that reflects current practices. The demonstrated method, however, can be applied on a rolling basis, so that facilities can assess their practices over time and evaluate any adjustments made along the way.

While the dataset comprises a large portion of U.S. hospitalizations, there could be data imbalances by state or other factors not considered in the study. This may affect our ability to measure associations with some variables with low counts, such as some of the states. However, this would not affect our results as long as the data imbalances are not directly related to the coding specificity. Also, the cohort definition includes only a subset of dementia-related codes (F ICD-10 diagnosis codes). A more expansive cohort definition is possible, but it would not affect the approach taken, since the cohort definition is common across facilities.

We utilize administrative claims data for explaining a substantial portion of the coding specificity variability in healthcare facilities. While this is insufficient to explain the full variability of diagnostic coding specificity, it is noteworthy that this explanatory power was achieved with minimal access to patients’ clinical characteristics, such as those provided in EHRs, many of which are not commonly available in claims data. This indicates that the model provides a baseline from which substantial improvements are possible if additional information is available, such as the granularity and clinical details found in EHRs. However, by making EHRs an optional input, our model gains generalizability, since there is no need for a clinical metric against which to measure the ‘correctness’ of the degree of coding specificity. Thus, while such clinical metric would be ideal, it is also unfeasible. Therefore, our approach should only be used as a metric to compare against industry standards and averages or against aspirational peer facilities.

Our approach assumes that patients are provided homogeneous treatments within facilities conditional on the set of variables used in the multivariate logistic regression. However, this assumption could be relaxed by introducing additional clustering factors/variables, such as the physicians within facilities, which may explain additional sources of coding specificity variability. The assumption of independence across hospitalizations could also be questionable, since there will be a substantial number of unmeasured factors that could contribute to a lack of independence (e.g., how busy the facilities were during the hospitalizations, who provided treatment, what the commonalities of the unmeasured clinical components across patients were, etc.). However, the model provides an initial metric to flag facilities with the potential for non-standard specificity practices, which can then be investigated more thoroughly by quality-control personnel.

The inclusion of random effects in the model was first considered across a range of facility-level characteristics, particularly the facility identifier. However, for the purpose of this study, we did not include random effects for multiple reasons as follows: (1) Computational complexity—for example, facility-specific random effects added hundreds of random effects in this particular dataset and potentially thousands or tens of thousands for other cohorts, leading to memory limitations. The proposed approach still required nearly 8 Gb RAM. Additionally, if even larger computational resources are needed, then the ability of quality-control personnel to use this approach could be substantially limited; (2) The reduced level of extrapolatability for even more complex or larger datasets, as administrative data may contain few observations or just one observation per facility, particularly if the tool is used for ‘live’ monitoring purposes; (3) Assumptions behind a random effects approach would be highly questionable, since the random effects would likely be correlated with some of the patient-level characteristics; and (4) While random effects and other modeling enhancements (e.g., different machine learning approaches or semiparametric models with spline components for some of the continuous variables, such as the log-length of stay) could have been considered for variables with lower numbers of categories, the purpose of our approach is not to find the optimal model for a particular cohort/year or set of variables. Instead, this manuscript aims to demonstrate the methodology and utility of administrative information in explaining diagnostic coding specificity variability among patients diagnosed with dementia. The purpose of the two-step approach (first at the patient visit level and then aggregated at any level) is to also provide tools that can be used in different forms, both in a disaggregated form for patient visit monitoring and in an aggregated form for facility monitoring.

The presence of multicollinearity among risk adjustment factors can complicate coefficient interpretation. Alternative approaches that map the information content to smaller sets of uncorrelated factors may be viable to reduce variance inflation, though they would be highly complex to construct due to the mostly categorical structure of the explanatory variable set. Such alternatives could also reduce interpretability. Multicollinearity, however, does not impact the main outcome of this manuscript, which is the estimation of a probability metric for coding specificity at the hospitalization level and a subsequent facility-level aggregation to measure facilities against healthcare industry standards. The goodness of fit or model use for prediction are not affected by collinearity, which allows for wide arrays of explanatory variables to be combined, regardless of potential information overlap in these variables. Thus, the focus of this manuscript is the metrics at the hospitalization and facility levels and their utility in identifying hospitalizations and facilities whose outcomes may substantially differ from industry practices, rather than the specific associations between explanatory variables and outcomes.

Finally, some quantitative data were provided in grouped categories for confidentiality purposes (e.g., age and bed size), and additional variables were not included to maintain the confidentiality of the records. This additional granularity and information could prove to enhance model outcomes within healthcare facility settings.

## 5. Conclusions

Medical coding is a very important component of healthcare systems, with an extensive impact on patient care quality, reimbursement, and system reliability. An understudied aspect of coding accuracy relates to coding specificity to the highest precision clinically possible. Our study focused on dementia coding specificity in the U.S. and demonstrates that a large number of readily available patient- and facility-level characteristics can be used to make risk adjustments to the odds of coding specificity and thus provide a standardized metric against which facilities can compare their coding specificity practices and standards. This study provides healthcare facilities with a valuable tool to enhance and assess variations in coding specificity, thus contributing to improved healthcare system reliability and financial efficiency as well as improved patient care in an era when accuracy and precision are of the utmost importance. The method demonstrated in this manuscript fills a significant gap in the literature, and its adaptability across patient cohorts, health conditions, and clusters of healthcare provision makes it a valuable tool for quality control and performance assessment. Our results indicate that the variability in the coding specificity of principal diagnoses of dementia can be better explained than the variability in the specificity of secondary diagnoses of dementia. This study addresses a critical need by making risk adjustments for factors that influence coding practices, ultimately contributing to our understanding of coding specificity disparities.

## Figures and Tables

**Table 1:** Descriptive statistics of the dementia-related principal and secondary diagnosis coding specificity outcomes as well as patient and facility characteristics (counts and means/proportions and corresponding percentages/standard deviations).

Study Variables	Count or Mean/Proportion (% or Standard Deviation (SD))
***Outcomes***	
Specificity of dementia principal diagnosis (count, proportion)	1788 (17%)
Specificity of dementia secondary diagnoses (count, proportion)	186,300 (39%)
***Patient Characteristics***	
*Age (Years)*	
0–44	809 (<1%)
45–54	2735 (1%)
55–59	5492 (1%)
60–64	13,512 (3%)
65–69	27,970 (6%)
70–74	53,037 (11%)
75–79	83,694 (17%)
80–84	103,805 (21%)
85+	196,721 (40%)
*Sex*	
Female	282,090 (58%)
Male	205,685 (42%)
*Race*	
Asian	12,539 (3%)
Black	68,784 (14%)
Other	26,675 (5%)
Unable to determine	10,463 (2%)
White	369,314 (76%)
*Log(Length of Stay) (Days) (mean, SD)*	1.6 (0.85)
*Primary Payor*	
Charity/Indigent	200 (<1%)
Commercial indemnity	4613 (1%)
Direct employer contract	217 (<1%)
Managed care capitated	417 (<1%)
Managed care non-capitated	9370 (2%)
Medicaid managed care capitated	1028 (<1%)
Medicaid managed care non-capitated	7807 (2%)
Medicaid traditional	5512 (1%)
Medicare managed care capitated	20,202 (4%)
Medicare managed care non-capitated	165,874 (34%)
Medicare traditional	258,584 (53%)
Other	2713 (1%)
Other government payors	9097 (2%)
Self-pay	1950 (<1%)
Workers’ compensation	191 (<1%)
*Point of Origin*	
Clinic	16,400 (3%)
Court/Law enforcement	215 (<1%)
Information not available	3174 (1%)
Non-healthcare facility	385,271 (79%)
Other	425 (<1%)
Transfer from ambulatory surgery center	2767 (1%)
Transfer from dept unit in same hospital, separate claim	302 (<1%)
Transfer from health facility	7337 (2%)
Transfer from hospice and under hospice care	131 (<1%)
Transfer from hospital (different facility)	27,141 (6%)
Transfer from SNF ^1^ or ICF ^2^	44,612 (9%)
*Discharge Status*	
Acute inpatient readmission	847 (<1%)
Discharged to home health organization	89,392 (18%)
Discharged to home or self-care	91,843 (19%)
Discharged to hospice home	25,625 (5%)
Discharged to hospice medical facility	24,993 (5%)
Discharged/Transferred to another rehab facility	15,425 (3%)
Discharged/Transferred to cancer center/children’s hospital	209 (<1%)
Discharged/Transferred to court/law enforcement	247 (<1%)
Discharged/Transferred to critical access hospital	71 (<1%)
Discharged/Transferred to federal hospital	279 (<1%)
Discharged/Transferred to ICF ^2^	12,891 (3%)
Discharged/Transferred to long-term care hospital	5448 (1%)
Discharged/Transferred to nursing facility	1732 (<1%)
Discharged/Transferred to other facility	5270 (1%)
Discharged/Transferred to other health institute not in list	1391 (<1%)
Discharged/Transferred to psychiatric hospital	2341 (<1%)
Discharged/Transferred to SNF ^1^	177,780 (36%)
Discharged/Transferred to swing bed	1605 (<1%)
Expired	28,217 (6%)
Information not available	232 (<1%)
Left against medical advice	1901 (<1%)
Still a patient—expected to return	36 (<1%)
*Count of Procedures (mean, SD)*	(2.7) 2.2
*CMS ^3^ Fiscal Year*	
2022	359,803 (74%)
2023	127,972 (26%)
*Social Vulnerability Indices (mean, SD)*	
Household characteristics	0.52% (0.25)
Housing type and transportation	0.62% (0.24)
Overall	0.61% (0.25)
Racial and ethnic minority status	0.71% (0.23)
Socioeconomic status	0.56% (0.26)
*COVID-19 Status*	
Not identified	425,762 (87%)
Positive	62,013 (13%)
*MS-DRG ^4^ Type*	
Medical	412,512 (85%)
Surgical	75,263 (15%)
***Facility Characteristics***	
*Teaching Status*	
No	378,768 (78%)
Not available	6221 (1%)
Yes	102,786 (21%)
*Academic Status*	
No	415,274 (85%)
Yes	72,501 (15%)
*Rural/Urban Status*	
Rural	66,204 (14%)
Urban	421,571 (86%)
*Ownership*	
Government—federal	1118 (<1%)
Government—hospital district/authority	32,417 (7%)
Government—local	11,798 (2%)
Government—state	3611 (1%)
Not available	2248 (<1%)
Physician	1032 (<1%)
Proprietary	25,459 (5%)
Voluntary non-profit—church	71,085 (15%)
Voluntary non-profit—other	26,015 (5%)
Voluntary non-profit—private	312,992 (64%)
*Bed Count*	
1–50	12,300 (3%)
51–100	25,881 (5%)
101–150	38,616 (8%)
151–200	32,908 (7%)
201–250	47,519 (10%)
251–300	50,418 (10%)
301–350	53,077 (11%)
351–400	39,018 (8%)
>400	188,038 (39%)
*Case Mix Index (mean, SD)*	1.76 (0.26)
*State Abbreviation*	
AK	253 (<1%)
AL	3966 (1%)
AR	3789 (1%)
AZ	12,029 (2%)
CA	27,281 (6%)
CO	3578 (1%)
CT	5982 (1%)
DE	1125 (<1%)
FL	60,713 (12%)
GA	7318 (2%)
HI	6038 (1%)
IA	4759 (1%)
ID	28 (<1%)
IL	18,082 (4%)
IN	6646 (1%)
KS	2856 (1%)
KY	10,899 (2%)
LA	3505 (1%)
MA	4549 (1%)
MD	7131 (1%)
ME	21 (<1%)
MI	22,526 (5%)
MN	3431 (1%)
MO	3382 (1%)
MS	6744 (1%)
MT	1374 (<1%)
NC	27,936 (6%)
ND	676 (<1%)
NE	1965 (<1%)
NH	57 (<1%)
NJ	9184 (2%)
NM	2360 (<1%)
NV	5537 (1%)
NY	45,302 (9%)
OH	22,769 (5%)
OK	7968 (2%)
OR	7750 (2%)
PA	24,500 (5%)
RI	15 (<1%)
SC	10,009 (2%)
SD	1194 (<1%)
TN	16,120 (3%)
TX	32,134 (7%)
UT	38 (<1%)
VA	16,695 (3%)
VT	163 (<1%)
WA	7833 (2%)
WI	9889 (2%)
WV	9472 (2%)
WY	204 (<1%)

^1^ SNF: Skilled nursing facility; ^2^ ICF: Intermediate care facility; ^3^ CMS: U.S. Centers for Medicare and Medicaid Services; ^4^ MS-DRG: Medicare Severity Diagnosis Related Group.

**Table 2:** Univariate and multivariate logistic regression results including odds ratios (ORs), corresponding 95% confidence intervals (CIs), and *p*-values for specificity of a dementia-related principal diagnosis.

		Univariate Analysis			Multivariate Analysis	
Variable	OR	95% CI	*p*-Value	OR	95% CI	*p*-Value
*Intercept*	-	-	-	0.030	0.016–0.057	<0.001
*Age (Ref: 85+)*						
0–44	5.698	2.111–15.378	0.001	2.599	0.781–8.646	0.119
45–54	5.976	3.679–9.706	<0.001	4.845	2.731–8.596	<0.001
55–59	3.337	2.276–4.894	<0.001	2.427	1.577–3.735	<0.001
60–64	3.281	2.546–4.227	<0.001	2.601	1.930–3.507	<0.001
65–69	2.722	2.254–3.288	<0.001	2.266	1.843–2.786	<0.001
70–74	1.583	1.330–1.884	<0.001	1.483	1.232–1.785	<0.001
75–79	1.726	1.482–2.009	<0.001	1.663	1.414–1.955	<0.001
80–84	1.556	1.338–1.809	<0.001	1.484	1.266–1.740	<0.001
*Sex (Ref: Female)*						
Male	1.591	1.436–1.763	<0.001	1.454	1.301–1.625	<0.001
*Race (Ref: White)*						
Asian	1.485	1.084–2.035	0.014	1.308	0.907–1.888	0.151
Black	1.385	1.213–1.581	<0.001	1.237	1.058–1.446	0.008
Other	1.215	0.986–1.498	0.068	0.907	0.713–1.154	0.428
Unable to determine	0.811	0.570–1.154	0.244	0.801	0.551–1.166	0.247
*Log(Length of Stay)*	1.272	1.212–1.336	<0.001	1.124	1.060–1.191	<0.001
*Primary Payor (Ref: Medicare traditional)*						
Charity/Indigent	1.305	0.146–11.694	0.812	0.462	0.038–5.576	0.543
Commercial indemnity	1.334	0.914–1.948	0.136	1.054	0.694–1.601	0.805
Direct employer contract	0.000	0.000-Inf	0.943	0.000	0.000-Inf	0.989
Managed care capitated	0.870	0.105–7.238	0.898	0.604	0.062–5.919	0.665
Managed care non-capitated	1.449	1.107–1.898	0.007	1.195	0.887–1.610	0.240
Medicaid managed care capitated	2.901	1.537–5.477	0.001	0.924	0.436–1.956	0.836
Medicaid managed care non-capitated	1.875	1.350–2.604	<0.001	1.083	0.738–1.590	0.684
Medicaid traditional	2.437	1.677–3.541	<0.001	1.236	0.802–1.907	0.337
Medicare managed care capitated	1.096	0.829–1.449	0.521	0.902	0.659–1.235	0.520
Medicare managed care non-capitated	1.091	0.972–1.224	0.140	1.048	0.924–1.190	0.463
Other	1.555	0.852–2.838	0.150	1.311	0.690–2.492	0.409
Other government payors	1.707	1.234–2.362	0.001	1.129	0.779–1.637	0.521
Self-pay	1.093	0.531–2.250	0.809	0.757	0.353–1.624	0.475
Workers’ compensation	2.611	0.236–28.827	0.434	1.416	0.105–19.117	0.793
*Point of Origin (Ref: Non-healthcare facility)*						
Clinic	1.339	0.996–1.799	0.053	1.375	0.994–1.902	0.055
Court/Law enforcement	1.788	0.569–5.623	0.320	1.394	0.411–4.724	0.594
Information not available	3.659	2.308–5.802	<0.001	3.949	2.321–6.719	<0.001
Other	1.639	0.17–15.769	0.669	1.473	0.141–15.383	0.746
Transfer from ambulatory surgery center	0.819	0.099–6.812	0.854	0.688	0.080–5.930	0.734
Transfer from dept unit in same hospital, separate claim	1.414	0.868–2.306	0.164	1.475	0.858–2.536	0.160
Transfer from health facility	0.922	0.556–1.530	0.753	1.001	0.586–1.709	0.997
Transfer from hospice and under hospice program	0.000	0.000-Inf	0.946	0.000	0.000-Inf	0.984
Transfer from hospital (different facility)	1.302	1.012–1.677	0.040	1.026	0.776–1.357	0.856
Transfer from SNF ^1^ or ICF ^2^	1.097	0.882–1.364	0.404	1.133	0.893–1.437	0.304
*Discharge Status (Ref: Discharged to home or self-care)*						
Acute inpatient readmission	0.716	0.213–2.404	0.588	0.840	0.238–2.961	0.786
Discharged to home health organization	1.106	0.929–1.316	0.257	1.144	0.948–1.382	0.161
Discharged to hospice home	1.202	0.921–1.568	0.176	1.367	1.023–1.828	0.035
Discharged to hospice medical facility	1.232	0.937–1.620	0.135	1.354	1.006–1.823	0.046
Discharged/Transferred to another rehab facility	1.131	0.785–1.628	0.510	1.250	0.845–1.848	0.264
Discharged/Transferred to court/law enforcement	15.741	1.633–151.780	0.017	3.375	0.293–38.812	0.329
Discharged/Transferred to federal hospital	2.099	0.460–10.862	0.377	2.089	0.368–11.873	0.406
Discharged/Transferred to ICF ^2^	1.218	0.913–1.624	0.180	1.142	0.831–1.570	0.414
Discharged/Transferred to long-term care hospital	0.562	0.280–1.129	0.105	0.519	0.244–1.104	0.089
Discharged/Transferred to nursing facility	1.331	0.772–2.295	0.303	1.185	0.636–2.209	0.594
Discharged/Transferred to other facility	1.088	0.637–1.857	0.758	1.132	0.641–2.000	0.669
Discharged/Transferred to other health institute not in list	1.199	0.552–2.608	0.646	1.217	0.531–2.790	0.642
Discharged/Transferred to psychiatric hospital	1.344	1.013–1.785	0.041	1.457	1.069–1.986	0.017
Discharged/Transferred to SNF ^1^	1.127	0.979–1.297	0.096	1.144	0.979–1.338	0.091
Discharged/Transferred to swing bed	1.166	0.251–5.420	0.845	1.227	0.234–6.440	0.809
Expired	1.282	0.901–1.824	0.168	0.991	0.672–1.462	0.965
Information not available	0.000	0.000-Inf	0.954	0.000	0.000-Inf	0.986
Left against medical advice	0.777	0.367–1.648	0.511	0.888	0.400–1.969	0.770
Still a patient—expected to return	0.000	0.000-Inf	0.962	0.000	0.000-Inf	0.989
*Count of Procedures*	1.145	1.109–1.183	<0.001	1.230	1.179–1.283	<0.001
*CMS ^3^ Fiscal Year (Ref: 2022)*						
2023	0.336	0.290–0.388	<0.001	0.264	0.224–0.311	<0.001
*Social Vulnerability Index*						
Household characteristics	1.506	1.238–1.831	<0.001	1.625	0.873–3.025	0.126
Housing type and transportation	1.329	1.080–1.636	0.007	1.362	0.584–3.177	0.474
Overall	1.326	1.085–1.621	0.006	0.361	0.037–3.561	0.383
Racial and ethnic minority status	1.047	0.830–1.321	0.698	0.829	0.491–1.398	0.482
Socioeconomic status	1.265	1.045–1.532	0.016	2.003	0.600–6.685	0.259
*COVID-19 Status (Ref: Not identified)*						
Positive	1.130	0.927–1.377	0.227	0.978	0.788–1.215	0.842
*MS-DRG ^4^ Type (Ref: Medical)*						
Surgical	2.089	1.482–2.943	<0.001	1.195	0.794–1.800	0.393
*Teaching Status (Ref: No)*						
Not Available	0.545	0.249–1.190	0.127	0.349	0.142–0.856	0.021
Yes	1.349	1.208–1.506	<0.001	1.047	0.839–1.307	0.685
*Academic Status (Ref: No)*						
Yes	1.270	1.124–1.435	<0.001	0.900	0.701–1.156	0.411
*Rural/Urban Status (Ref: Urban)*						
Rural	0.910	0.775–1.068	0.249	0.995	0.803–1.232	0.963
*Ownership (Ref: Voluntary non-profit—private)*						
Government—federal	0.000	0.000-Inf	0.934	0.000	0.000-Inf	0.981
Government—hospital district/authority	0.945	0.763–1.170	0.602	0.847	0.652–1.101	0.214
Government—local	1.591	1.166–2.170	0.003	2.104	1.401–3.159	<0.001
Government—state	0.718	0.389–1.323	0.228	0.641	0.304–1.352	0.243
Not available	1.871	0.774–4.522	0.164	1.758	0.679–4.553	0.245
Physician	2.272	0.206–25.082	0.503	3.229	0.267–39.096	0.357
Proprietary	0.943	0.762–1.166	0.586	0.984	0.752–1.289	0.908
Voluntary non-profit—church	0.782	0.661–0.925	0.004	0.869	0.711–1.063	0.173
Voluntary non-profit—other	0.723	0.545–0.958	0.024	0.605	0.442–0.827	0.002
*Bed Count (Ref: >400)*						
1–50	0.605	0.403–0.908	0.015	0.687	0.426–1.107	0.123
51–100	0.592	0.460–0.760	<0.001	0.671	0.492–0.916	0.012
101–150	0.795	0.649–0.975	0.028	0.842	0.652–1.086	0.185
151–200	0.656	0.514–0.838	0.001	0.706	0.530–0.939	0.017
201–250	0.619	0.510–0.751	<0.001	0.818	0.645–1.037	0.097
251–300	0.603	0.501–0.725	<0.001	0.704	0.559–0.886	0.003
301–350	0.727	0.604–0.876	0.001	0.865	0.688–1.086	0.212
351–400	0.754	0.608–0.935	0.010	0.933	0.718–1.211	0.601
*Case Mix Index*	1.912	1.574–2.322	<0.001	1.579	1.188–2.100	0.002
*State Abbreviation (Ref: NY)*						
AK	2.708	0.245–29.975	0.417	4.569	0.382–54.824	0.230
AL	1.504	0.836–2.707	0.713	1.187	0.625–2.254	0.600
AR	0.524	0.224–1.224	0.136	0.413	0.163–1.044	0.062
AZ	0.633	0.313–1.279	0.203	0.554	0.265–1.157	0.116
CA	1.216	0.931–1.588	0.152	1.242	0.901–1.714	0.186
CO	0.478	0.146–1.568	0.223	0.470	0.316–1.621	0.232
CT	1.658	0.916–3.002	0.095	1.770	0.934–3.352	0.080
DE	0.000	0.000-Inf	0.952	0.000	0.000-Inf	0.966
FL	0.928	0.748–1.152	0.500	0.860	0.654–1.130	0.279
GA	1.511	0.943–2.422	0.860	0.874	0.492–1.556	0.648
HI	1.884	1.050–3.379	0.034	2.239	1.019–4.920	0.045
IA	1.625	0.944–2.798	0.080	1.256	0.676–2.336	0.471
IL	0.668	0.465–0.960	0.029	0.644	0.428–0.696	0.035
IN	1.236	0.755–2.023	0.399	1.067	0.621–1.835	0.814
KS	1.489	0.754–2.941	0.251	0.879	0.412–1.873	0.738
KY	1.146	0.751–1.749	0.526	1.017	0.621–1.667	0.945
LA	4.431	2.343–8.379	<0.001	2.794	1.358–5.748	0.005
MA	1.389	0.957–2.015	0.084	1.256	0.819–1.928	0.297
MD	0.931	0.581–1.492	0.766	1.103	0.653–1.862	0.714
ME	0.000	0.000-Inf	0.984	0.000	0.000-Inf	0.989
MI	0.947	0.726–1.236	0.690	0.840	0.599–1.178	0.313
MN	1.743	1.115–2.724	0.015	1.842	1.127–3.01	0.015
MO	0.602	0.237–1.531	0.286	0.362	0.128–1.021	0.055
MS	0.733	0.405–1.329	0.307	0.582	0.306–1.109	0.100
MT	0.226	0.030–1.676	0.146	0.148	0.018–1.205	0.074
NC	1.645	1.282–2.111	<0.001	1.101	0.796–1.523	0.561
ND	2.407	0.736–7.875	0.146	3.664	0.972–13.816	0.055
NE	0.492	0.150–1.617	0.243	0.661	0.912–2.277	0.512
NJ	0.961	0.700–1.318	0.805	0.988	0.692–1.413	0.949
NM	1.444	0.753–2.768	0.268	1.513	0.736–3.113	0.260
NV	0.782	0.515–1.187	0.248	0.906	0.542–1.513	0.705
OH	1.287	1.001–1.654	0.049	1.077	0.791–1.467	0.637
OK	1.281	0.910–1.802	0.156	1.044	0.676–1.613	0.845
OR	2.462	1.567–3.868	<0.001	2.552	1.530–4.257	<0.001
PA	2.003	1.623–2.472	<0.001	2.146	1.656–2.782	<0.001
SC	1.044	0.699–1.559	0.833	0.927	0.592–1.450	0.739
SD	0.000	0.000-Inf	0.944	0.000	0.000-Inf	0.962
TN	0.752	0.534–1.059	0.102	0.598	0.399–0.897	0.013
TX	0.974	0.696–1.361	0.876	0.929	0.627–1.374	0.711
UT	0.000	0.000-Inf	0.988	0.000	0.000-Inf	0.992
VA	1.477	1.097–1.989	0.010	1.506	1.052–2.155	0.025
VT	0.000	0.000-Inf	0.977	0.000	0.000-Inf	0.986
WA	0.896	0.554–1.448	0.653	0.965	0.562–1.656	0.897
WI	1.529	1.026–2.276	0.037	1.500	0.952–2.363	0.080
WV	0.921	0.590–1.437	0.717	0.726	0.434–1.216	0.224
WY	0.000	0.000-Inf	0.974	0.000	0.000-Inf	0.982

^1^ SNF: Skilled nursing facility; ^2^ ICF: Intermediate care facility; ^3^ CMS: U.S. Centers for Medicare and Medicaid Services; ^4^ MS-DRG: Medicare Severity Diagnosis Related Group.

**Table 3:** Univariate and multivariate logistic regression results including odds ratios (ORs), corresponding 95% confidence intervals (CIs), and *p*-values for specificity of a dementia-related secondary diagnosis.

		Univariate Analysis			Multivariate Analysis	
Variable	OR	95% CI	*p*-Value	OR	95% CI	*p*-Value
*Intercept*	-	-	-	0.351	0.327–0.378	<0.001
*Age (Ref: 85+)*						
0–44	1.956	1.701–2.248	<0.001	1.934	1.676–2.233	<0.001
45–54	1.736	1.607–1.875	<0.001	1.734	1.601–1.877	<0.001
55–59	1.745	1.652–1.843	<0.001	1.736	1.640–1.838	<0.001
60–64	1.532	1.477–1.588	<0.001	1.526	1.468–1.586	<0.001
65–69	1.459	1.421–1.497	<0.001	1.422	1.384–1.461	<0.001
70–74	1.438	1.409–1.467	<0.001	1.421	1.392–1.451	<0.001
75–79	1.410	1.386–1.435	<0.001	1.402	1.377–1.426	<0.001
80–84	1.316	1.295–1.337	<0.001	1.316	1.295–1.338	<0.001
*Sex (Ref: Female)*						
Male	1.269	1.254–1.284	<0.001	1.224	1.209–1.239	<0.001
*Race (Ref: White)*						
Asian	0.977	0.941–1.015	0.230	1.009	0.967–1.053	0.680
Black	0.989	0.972–1.006	0.202	0.955	0.937–0.973	<0.001
Other	1.036	1.009–1.063	0.008	1.069	1.040–1.099	<0.001
Unable to determine	0.969	0.930–1.010	0.139	0.973	0.933–1.015	0.210
*Log(Length of Stay)*	1.069	1.062–1.077	<0.001	1.017	1.008–1.025	<0.001
*Primary Payor (Ref: Medicare traditional)*						
Charity/Indigent	1.204	0.903–1.604	0.206	0.976	0.729–1.308	0.873
Commercial indemnity	1.121	1.055–1.192	<0.001	0.979	0.919–1.042	0.499
Direct employer contract	2.934	2.229–3.861	<0.001	2.449	1.854–3.234	<0.001
Managed care capitated	1.087	0.889–1.328	0.416	0.863	0.703–1.060	0.161
Managed care non-capitated	1.090	1.044–1.139	<0.001	1.011	0.967–1.057	0.639
Medicaid managed care capitated	1.130	0.993–1.285	0.063	0.896	0.785–1.023	0.105
Medicaid managed care non-capitated	1.129	1.077–1.183	<0.001	0.896	0.852–0.942	<0.001
Medicaid traditional	1.050	0.993–1.111	0.087	0.844	0.796–0.896	<0.001
Medicare managed care capitated	1.018	0.987–1.049	0.258	0.979	0.948–1.012	0.214
Medicare managed care non-capitated	0.973	0.961–0.986	<0.001	0.945	0.933–0.958	<0.001
Other	0.963	0.888–1.043	0.354	0.909	0.837–0.986	0.022
Other government payors	1.081	1.034–1.129	0.001	0.915	0.875–0.958	<0.001
Self-pay	0.879	0.798–0.968	0.009	0.819	0.742–0.904	<0.001
Workers’ compensation	0.467	0.327–0.667	<0.001	0.495	0.344–0.711	<0.001
*Point of Origin (Ref: Non-healthcare facility)*						
Clinic	0.923	0.893–0.955	<0.001	0.954	0.921–0.987	0.007
Court/Law enforcement	1.029	0.771–1.372	0.848	0.949	0.698–1.291	0.739
Information not available	1.008	0.936–1.085	0.838	1.045	0.968–1.129	0.256
Other	1.071	0.878–1.306	0.497	1.159	0.946–1.419	0.154
Transfer from ambulatory surgery center	0.769	0.599–0.988	0.040	0.715	0.555–0.921	0.009
Transfer from dept unit in same hospital, separate claim	0.976	0.901–1.057	0.543	0.999	0.921–1.084	0.982
Transfer from health facility	1.003	0.956–1.053	0.896	0.950	0.904–0.999	0.044
Transfer from hospice and under hospice program	1.288	0.904–1.834	0.161	1.248	0.871–1.787	0.227
Transfer from hospital (different facility)	0.950	0.926–0.975	<0.001	0.884	0.860–0.908	<0.001
Transfer from SNF ^1^ or ICF ^2^	1.118	1.096–1.141	<0.001	1.082	1.059–1.106	<0.001
*Discharge Status (Ref: Discharged to home or self-care)*						
Acute inpatient readmission	0.966	0.835–1.117	0.637	1.033	0.891–1.197	0.666
Discharged to home health organization	1.026	1.006–1.046	0.011	1.064	1.043–1.085	<0.001
Discharged to hospice home	1.239	1.204–1.275	<0.001	1.312	1.274–1.352	<0.001
Discharged to hospice medical facility	1.180	1.146–1.215	<0.001	1.228	1.191–1.265	<0.001
Discharged/Transferred to another rehab facility	1.010	0.974–1.047	0.589	1.019	0.982–1.058	0.321
Discharged/Transferred to cancer ctr/children’s hospital	0.938	0.702–1.253	0.666	1.109	0.827–1.488	0.490
Discharged/Transferred to court/law enforcement	1.249	0.965–1.616	0.092	1.128	0.856–1.486	0.392
Discharged/Transferred to critical access hospital	0.612	0.355–1.056	0.078	0.581	0.335–1.008	0.054
Discharged/Transferred to federal hospital	1.027	0.800–1.319	0.833	1.027	0.797–1.322	0.839
Discharged/Transferred to ICF ^2^	1.289	1.240–1.339	<0.001	1.249	1.201–1.300	<0.001
Discharged/Transferred to long-term care hospital	1.108	1.046–1.173	<0.001	1.002	0.944–1.063	0.950
Discharged/Transferred to nursing facility	1.532	1.389–1.690	<0.001	1.544	1.396–1.706	<0.001
Discharged/Transferred to other facility	0.978	0.922–1.038	0.463	0.947	0.891–1.005	0.074
Discharged/Transferred to other health institute not in list	1.189	1.064–1.328	0.002	1.196	1.068–1.338	0.002
Discharged/Transferred to psychiatric hospital	1.849	1.694–2.019	<0.001	1.685	1.542–1.842	<0.001
Discharged/Transferred to SNF ^1^	1.050	1.032–1.068	<0.001	1.075	1.056–1.095	<0.001
Discharged/Transferred to swing bed	1.084	0.977–1.202	0.127	1.151	1.034–1.280	0.010
Expired	0.936	0.910–0.963	<0.001	0.896	0.870–0.923	<0.001
Information not available	0.691	0.514–0.928	0.014	0.830	0.613–1.124	0.228
Left against medical advice	0.850	0.769–0.940	0.002	0.829	0.749–0.917	<0.001
Still a patient—expected to return	1.204	0.603–2.405	0.598	1.006	0.498–2.034	0.987
*Count of Procedures*	1.038	1.036–1.041	<0.001	1.039	1.036–1.042	0.001
*CMS ^3^ Fiscal Year (Ref: 2022)*						
2023	1.030	1.016–1.044	<0.001	1.014	1.000–1.028	0.049
*Social Vulnerability Index*						
Household characteristics	0.808	0.790–0.827	<0.001	0.829	0.717–0.958	0.011
Housing type and transportation	0.936	0.914–0.958	<0.001	0.963	0.892–1.040	0.336
Overall	0.912	0.889–0.936	<0.001	1.090	1.030–1.154	0.003
Racial and ethnic minority status	0.911	0.889–0.934	<0.001	0.931	0.844–1.027	0.156
Socioeconomic status	0.836	0.817–0.857	<0.001	1.177	0.895–1.548	0.245
*COVID-19 Status (Ref: Not identified)*						
Positive	0.960	0.943–0.977	<0.001	0.948	0.930–0.965	<0.001
*MS-DRG ^4^ Type (Ref: Medical)*						
Surgical	0.932	0.917–0.947	<0.001	0.859	0.844–0.875	<0.001
*Teaching Status (Ref: No)*						
Not Available	1.153	1.095–1.215	<0.001	1.130	1.066–1.197	<0.001
Yes	1.067	1.051–1.082	<0.001	0.975	0.949–1.001	0.061
*Academic Status (Ref: No)*						
Yes	1.078	1.061–1.096	<0.001	1.052	1.020–1.085	<0.001
*Rural/Urban Status (Ref: Urban)*						
Rural	0.969	0.952–0.986	<0.001	0.976	0.955–0.997	0.025
*Ownership (Ref: Voluntary non-profit—private)*						
Government—federal	0.952	0.841–1.078	0.437	0.915	0.802–1.042	0.181
Government—hospital district/authority	1.048	1.024–1.074	<0.001	1.033	1.006–1.062	0.017
Government—local	1.102	1.060–1.145	<0.001	1.146	1.097–1.196	<0.001
Government—state	0.898	0.837–0.963	0.003	0.881	0.815–0.951	0.001
Not available	0.905	0.829–0.989	0.028	0.847	0.771–0.930	0.001
Physician	1.364	1.206–1.544	<0.001	1.191	1.045–1.358	0.009
Proprietary	0.837	0.814–0.860	<0.001	0.872	0.845–0.899	<0.001
Voluntary non-profit—church	0.886	0.871–0.902	<0.001	0.904	0.887–0.921	<0.001
Voluntary non-profit—other	0.961	0.935–0.987	0.003	0.890	0.864–0.916	<0.001
*Bed Count (Ref: >400)*						
1–50	0.940	0.904–0.976	0.002	0.954	0.912–0.997	0.038
51–100	1.035	1.007–1.064	0.001	1.071	1.037–1.105	<0.001
101–150	0.849	0.829–0.869	<0.001	0.878	0.987–1.040	<0.001
151–200	0.952	0.928–0.975	<0.001	0.951	0.854–0.902	<0.001
201–250	0.868	0.849–0.886	<0.001	0.931	0.924–0.978	<0.001
251–300	0.914	0.895–0.933	<0.001	0.945	0.909–0.955	<0.001
301–350	0.908	0.889–0.926	<0.001	0.939	0.922–0.969	<0.001
351–400	0.924	0.903–0.945	<0.001	1.013	0.917–0.961	0.329
*Case Mix Index*	1.028	1.005–1.052	0.018	0.981	0.951–1.012	0.227
*State Abbreviation (Ref: NY)*						
AK	1.392	1.081–1.793	0.010	1.335	1.034–1.725	0.027
AL	0.731	0.679–0.787	<0.001	0.723	0.669–0.780	<0.001
AR	0.626	0.579–0.677	<0.001	0.665	0.612–0.722	<0.001
AZ	1.011	0.968–1.055	0.633	1.039	0.992–1.089	0.104
CA	0.946	0.916–0.977	0.001	0.949	0.915–0.984	0.005
CO	0.857	0.795–0.923	<0.001	0.841	0.778–0.909	<0.001
CT	1.240	1.173–1.312	<0.001	1.235	1.166–1.309	<0.001
DE	0.817	0.716–0.932	0.003	0.825	0.720–0.945	0.005
FL	0.912	0.888–0.936	<0.001	0.922	0.894–0.952	<0.001
GA	0.933	0.885–0.984	0.011	0.881	0.829–0.935	<0.001
HI	1.150	1.087–1.217	<0.001	1.219	1.136–1.309	<0.001
IA	1.342	1.261–1.427	<0.001	1.298	1.216–1.386	<0.001
ID	1.307	0.612–2.792	0.489	1.488	0.693–3.197	0.308
IL	1.061	1.023–1.101	0.002	1.087	1.044–1.131	<0.001
IN	1.093	1.035–1.154	0.001	1.095	1.033–1.161	0.002
KS	0.697	0.639–0.760	<0.001	0.685	0.626–0.750	<0.001
KY	1.196	1.144–1.249	<0.001	1.225	1.167–1.287	<0.001
LA	1.330	1.239–1.428	<0.001	1.287	1.194–1.388	<0.001
MA	1.025	0.960–1.095	0.454	0.962	0.899–1.031	0.275
MD	0.969	0.918–1.023	0.254	0.950	0.897–1.007	0.083
ME	1.243	0.515–3.000	0.628	1.372	0.563–3.347	0.487
MI	1.006	0.972–1.041	0.741	1.027	0.987–1.069	0.182
MN	2.162	2.014–2.321	<0.001	2.077	1.930–2.234	<0.001
MO	0.991	0.919–1.068	0.809	0.902	0.833–0.977	0.012
MS	1.005	0.951–1.062	0.861	0.992	0.936–1.052	0.796
MT	0.798	0.708–0.900	<0.001	0.848	0.749–0.960	0.009
NC	1.305	1.265–1.347	<0.001	1.244	1.199–1.290	<0.001
ND	1.495	1.280–1.746	<0.001	1.723	1.469–2.022	<0.001
NE	1.667	1.521–1.827	<0.001	1.808	1.643–1.990	<0.001
NH	0.483	0.250–0.933	0.030	0.514	0.265–0.996	0.049
NJ	0.863	0.821–0.907	<0.001	0.874	0.830–0.921	<0.001
NM	0.880	0.803–0.963	0.006	0.839	0.763–0.921	<0.001
NV	0.793	0.745–0.845	<0.001	0.873	0.815–0.936	<0.001
OH	1.287	1.244–1.331	<0.001	1.356	1.306–1.408	<0.001
OK	1.407	1.339–1.478	<0.001	1.350	1.277–1.426	<0.001
OR	1.756	1.672–1.845	<0.001	1.881	1.784–1.984	<0.001
PA	1.403	1.358–1.450	<0.001	1.439	1.389–1.492	<0.001
RI	3.030	1.078–8.516	0.035	2.763	0.977–7.813	0.055
SC	1.082	1.033–1.133	0.001	1.080	1.028–1.135	0.002
SD	1.409	1.252–1.586	<0.001	1.393	1.233–1.573	<0.001
TN	0.807	0.776–0.840	<0.001	0.826	0.790–0.865	<0.001
TX	1.239	1.203–1.277	<0.001	1.247	1.203–1.292	<0.001
UT	0.855	0.422–1.730	0.663	0.881	0.433–1.789	0.726
VA	1.374	1.324–1.426	<0.001	1.365	1.309–1.422	<0.001
VT	0.797	0.564–1.127	0.199	0.853	0.601–1.211	0.373
WA	1.225	1.165–1.288	<0.001	1.281	1.215–1.352	<0.001
WI	1.509	1.443–1.578	<0.001	1.496	1.426–1.569	<0.001
WV	0.971	0.926–1.019	0.229	0.981	0.930–1.036	0.492
WY	1.416	1.067–1.879	0.016	1.471	1.104–1.960	0.008

^1^ SNF: Skilled nursing facility; ^2^ ICF: Intermediate care facility; ^3^ CMS: Centers for Medicare and Medicaid Services; ^4^ MS-DRG: Medicare Severity Diagnosis Related Group.

## Data Availability

Data were provided by Premier, Inc. and can be requested via https://www.pinc-ai.com/.

## Supplementary Materials

The following supporting information can be downloaded at: https://www.mdpi.com/article/10.3390/healthcare12100983/s1, Table S1: List of specified dementia ICD-10 codes and corresponding descriptions; Table S2: List of unspecified dementia ICD-10 codes and corresponding descriptions.
