# Supplementary material for: A Data-Driven Approach to Defining Risk-Adjusted Coding Specificity Metrics for a Large U.S. Dementia Patient Cohort
Source: Healthcare (Basel). 2024 May 10;12(10):983. doi: 10.3390/healthcare12100983 (PMC11120868; doi:10.3390/healthcare12100983)
Supplement: Supplementary file 1 [file healthcare-12-00983-s001.zip › healthcare-2959241-supplementary.pdf]

Supplementary Table S1: List of specified dementia ICD-10 codes and corresponding descriptions

Specified ICD-10 Code	Description
F0150	Vascular dementia, unspecified severity, without behavioral disturbance, psychotic disturbance, mood disturbance, and anxiety
F0151	Vascular dementia with behavioral disturbance
F01511	Vascular dementia, unspecified severity, with agitation
F01518	Vascular dementia, unspecified severity, with other behavioral disturbance
F0152	Vascular dementia, unspecified severity, with psychotic disturbance
F0153	Vascular dementia, unspecified severity, with mood disturbance
F0154	Vascular dementia, unspecified severity, with anxiety
F01A0	Vascular dementia, mild, without behavioral disturbance, psychotic disturbance, mood disturbance, and anxiety
F01A11	Vascular dementia, mild, with agitation
F01A18	Vascular dementia, mild, with other behavioral disturbance
F01A2	Vascular dementia, mild, with psychotic disturbance
F01A3	Vascular dementia, mild, with mood disturbance
F01A4	Vascular dementia, mild, with anxiety
F01B0	Vascular dementia, moderate, without behavioral disturbance, psychotic disturbance, mood disturbance, and anxiety
F01B11	Vascular dementia, moderate, with agitation
F01B18	Vascular dementia, moderate, with other behavioral disturbance
F01B2	Vascular dementia, moderate, with psychotic disturbance
F01B3	Vascular dementia, moderate, with mood disturbance
F01B4	Vascular dementia, moderate, with anxiety
F01C0	Vascular dementia, severe, without behavioral disturbance, psychotic disturbance, mood disturbance, and anxiety
F01C11	Vascular dementia, severe, with agitation
F01C18	Vascular dementia, severe, with other behavioral disturbance
F01C2	Vascular dementia, severe, with psychotic disturbance
F01C3	Vascular dementia, severe, with mood disturbance
F01C4	Vascular dementia, severe, with anxiety
F0280	Dementia in other diseases classified elsewhere, unspecified severity, without behavioral disturbance, psychotic disturbance, mood disturbance, and anxiety
F0281	Dementia in other diseases classified elsewhere with behavioral disturbance
F02811	Dementia in other diseases classified elsewhere, unspecified severity, with agitation
F02818	Dementia in other diseases classified elsewhere, unspecified severity, with other behavioral disturbance

F0282	Dementia in other diseases classified elsewhere, unspecified severity, with psychotic disturbance
F0283	Dementia in other diseases classified elsewhere, unspecified severity, with mood disturbance
F0284	Dementia in other diseases classified elsewhere, unspecified severity, with anxiety
F02A0	Dementia in other diseases classified elsewhere, mild, without behavioral disturbance, psychotic disturbance, mood disturbance, and anxiety
F02A11	Dementia in other diseases classified elsewhere, mild, with agitation
F02A18	Dementia in other diseases classified elsewhere, mild, with other behavioral disturbance
F02A2	Dementia in other diseases classified elsewhere, mild, with psychotic disturbance
F02A3	Dementia in other diseases classified elsewhere, mild, with mood disturbance
F02A4	Dementia in other diseases classified elsewhere, mild, with anxiety
F02B0	Dementia in other diseases classified elsewhere, moderate, without behavioral disturbance, psychotic disturbance, mood disturbance, and anxiety
F02B11	Dementia in other diseases classified elsewhere, moderate, with agitation
F02B18	Dementia in other diseases classified elsewhere, moderate, with other behavioral disturbance
F02B2	Dementia in other diseases classified elsewhere, moderate, with psychotic disturbance
F02B3	Dementia in other diseases classified elsewhere, moderate, with mood disturbance
F02B4	Dementia in other diseases classified elsewhere, moderate, with anxiety
F02C0	Dementia in other diseases classified elsewhere, severe, without behavioral disturbance, psychotic disturbance, mood disturbance, and anxiety
F02C11	Dementia in other diseases classified elsewhere, severe, with agitation
F02C18	Dementia in other diseases classified elsewhere, severe, with other behavioral disturbance
F02C2	Dementia in other diseases classified elsewhere, severe, with psychotic disturbance
F02C3	Dementia in other diseases classified elsewhere, severe, with mood disturbance
F02C4	Dementia in other diseases classified elsewhere, severe, with anxiety

Supplementary Table S2: List of unspecified dementia ICD-10 codes and corresponding descriptions

**Unspecified ICD-10 Code**	**Description**
F0390	Unspecified dementia, unspecified severity, without behavioral disturbance, psychotic disturbance, mood disturbance, and anxiety
F0391	Unspecified dementia with behavioral disturbance
F03911	Unspecified dementia, unspecified severity, with agitation
F03918	Unspecified dementia, unspecified severity, with other behavioral disturbance
F0392	Unspecified dementia, unspecified severity, with psychotic disturbance
F0393	Unspecified dementia, unspecified severity, with mood disturbance
F0394	Unspecified dementia, unspecified severity, with anxiety
F03A0	Unspecified dementia, mild, without behavioral disturbance, psychotic disturbance, mood disturbance, and anxiety
F03A11	Unspecified dementia, mild, with agitation
F03A18	Unspecified dementia, mild, with other behavioral disturbance
F03A2	Unspecified dementia, mild, with psychotic disturbance
F03A3	Unspecified dementia, mild, with mood disturbance
F03A4	Unspecified dementia, mild, with anxiety
F03B0	Unspecified dementia, moderate, without behavioral disturbance, psychotic disturbance, mood disturbance, and anxiety
F03B11	Unspecified dementia, moderate, with agitation
F03B18	Unspecified dementia, moderate, with other behavioral disturbance
F03B2	Unspecified dementia, moderate, with psychotic disturbance
F03B3	Unspecified dementia, moderate, with mood disturbance
F03B4	Unspecified dementia, moderate, with anxiety
F03C0	Unspecified dementia, severe, without behavioral disturbance, psychotic disturbance, mood disturbance, and anxiety
F03C11	Unspecified dementia, severe, with agitation
F03C18	Unspecified dementia, severe, with other behavioral disturbance
F03C2	Unspecified dementia, severe, with psychotic disturbance
F03C3	Unspecified dementia, severe, with mood disturbance
F03C4	Unspecified dementia, severe, with anxiety
